# Community-Level Social Capital and Psychological Distress among the Elderly in Japan: A Population-Based Study

**DOI:** 10.1371/journal.pone.0142629

**Published:** 2015-11-09

**Authors:** Tomoko Kobayashi, Etsuji Suzuki, Masayuki Noguchi, Ichiro Kawachi, Soshi Takao

**Affiliations:** 1 Public Health, Department of Social Medicine, Graduate School of Medicine, Osaka University, Osaka, Japan; 2 Department of Epidemiology, Graduate School of Medicine, Dentistry and Pharmaceutical Sciences, Okayama University, Okayama, Japan; 3 Okayama Prefectural Mental Health and Welfare Center, Okayama, Japan; 4 Department of Social and Behavioral Sciences, Harvard T.H. Chan School of Public Health, Boston, Massachusetts, United States of America; Aichi Cancer Center Research Institute, JAPAN

## Abstract

Despite accumulating evidence, previous studies have not clearly separated the contribution of community-level social capital on mental health from that of individual-level social support. We examined the association between community-level social capital and psychological distress in a sample of older Japanese individuals, taking into account the effects of individual-level social capital and social support. We collected data via a cross-sectional survey among all residents aged ≥65 in three rural municipalities in Okayama Prefecture. We measured two components of social capital in the questionnaire: perceptions of trust and reciprocity in the community. Community-level social capital was obtained by aggregating individual responses and calculating the proportion of subjects reporting mistrust and lack of reciprocity. Psychological distress was assessed by the Kessler Psychological Distress scale. We calculated risk ratios (RRs) and 95% confidence intervals (CIs) for psychological distress using two-level Poisson regression models (9,761 individuals nested within 35 communities). The prevalence of psychological distress was 39.8%. Low community-level social capital was associated with psychological distress, even after controlling for individual-level social support, age, sex, educational attainment, frequency of alcohol consumption, smoking status, body mass index, marital status, socioeconomic status, and number of cohabiters. The adjusted RRs per 10% increase of the proportion of mistrust and lack of reciprocity in the communities were 1.23 (95% CI: 1.01–1.51) and 1.12 (95% CI: 1.02–1.24), respectively. Lower levels of community-level social capital are associated with psychological distress among the Japanese elderly population, even after adjusting for individual-level perceptions of social capital and social support.

## Introduction

According to the 2010 Global Burden of Disease Study [1], major depressive disorder is the second leading cause of disability in the world. Furthermore, mental health is an important public health issue in the context of aging societies. For example, of the total disease burden among those aged over 60, 1.3% (7.5 million disability adjusted life years) is attributed to major depressive disorders [2]. In addition, the number of elderly Japanese patients with mood disorder and depressions has increased in the past 15 years (158,000 in 1996 vs. 278,000 in 2011) [3]. In Japan, 25.9% of the population was aged ≥65 years in 2014 [4], and projection show that one in three Japanese will be aged ≥65 years by 2030 [5]. In the context of this rapid population aging, there is a growing interest in promoting healthy aging, including the mental health of the elderly.

Social capital has been identified as an important social determinant of mental health. Social capital is formally defined as the resources that individuals can access via their social networks [6]. These resources can take the form of trust between actors in a network, the exchange of information, instrumental support, emotional support, as well as social reinforcement. The concept of social capital has been analyzed at both the individual level as well as at the group level. Most previous studies have shown that individual perceptions of community social capital (e.g. perceptions about the trustworthiness of others in the network) are associated with better mental health [7–9]. However the impact of community-level social capital has been much less consistently documented [9], although more recent evidence tends to support the beneficial effects of community-level social capital for mental health [10–13]. Despite accumulating evidence on social capital and mental health, studies conducted among the elderly remain scarce at both individual and community levels. To our knowledge, evidence has been limited to individual-level data regarding depression [14, 15], depressive symptoms [16, 17], and psychological distress [15, 18]. Although one cross-sectional study in Japan showed that high community-level social capital was associated with better mental health [12], the impact of social capital on mental health among the elderly remains less clear. Indeed, further studies are needed to clarify these associations because the number of elderly patients with mental illness is increasing in Japan.

On a related issue, there has been debate as to whether social capital simply represents the re-labelling of “social support” [19, 20]. However, unlike social support, the utility of social capital lies in redirecting the focus on the collective social entity (e.g. neighborhoods or workplaces) rather than individuals [21, 22]. Social capital research in public health field has been influenced by the early theorists such as Bourdieu, Coleman, and Putnam. As a result, different researchers’ orientation toward social capital have tended to emphasize two distinct dimensions, i.e. social capital as social cohesion (the communitarian perspective) versus social capital as “resources derived from social networks” [23]. To our knowledge, few studies have examined these two approaches simultaneously, and a more comprehensive approach would provide greater understanding how they influence psychological distress together. In this context, it is worth examining the independent contribution of “community-level” social capital on mental health beyond individual-level social capital and social support. In other words, we examined both aspects of social capital (i.e., the material/supportive resources extracted from social networks), as well as both the individual- and community-level characteristics of social capital.

The aim of this research is to evaluate the relationship between community-level social capital and psychological distress, taking account of the effects of individual-level social capital and social support.

## Methods

### Ethics statement

The cover of the questionnaire gave a thorough explanation of the aim of the survey. If residents did not agree to participate in this survey, they could freely choose not to respond without any consequences. Thus, we considered the receipt of a completed questionnaire to indicate informed consent. We obtained the data from the Okayama Prefectural Government after the removal of personal identifiers and analyzed the data anonymously. The study was reviewed and approved by the Ethics Committee of the Okayama University Graduate School of Medicine, Dentistry and Pharmaceutical Sciences.

### Participants

The present study was based on the Okayama Mental Health Survey of Elderly People, conducted by the Prefecture Government of Okayama in August 2010 [24–27]. Questionnaires were mailed to all residents (*n* = 21,232) aged 65 or over in three rural municipalities in Okayama Prefecture (located in the western part of Japan). The areal unit in our multilevel analysis was based on the administrative boundaries for public health service, and categorized into 35 communities (the mean number of residents in a community: 1,777, standard deviation: 1,241). Two hundred and one subjects were considered ineligible because their address was unknown or they were deceased. Of the 13,929 subjects who returned questionnaires (response rate: 65.6%), we excluded subjects who did not respond to questionnaire items regarding age, sex, address, social capital, and any items concerning psychological distress. Furthermore, we excluded residents who had lived in the communities for less than five years to avoid contamination from the possible effect of social capital of their previous residential communities. A total of 9,761 subjects were included in the analysis.

### Measures

#### Social capital

We measured two components of social capital based on individual responses to the survey questionnaire. The first item concerned general trust in the community: “Generally speaking, would you say that most people in your community can be trusted?” Participants could answer “1 = can be trusted”, “2 = can be somewhat trusted”, “3 = neither”, “4 = cannot be somewhat trusted”, or “5 = cannot be trusted”. The second item concerned reciprocity: “Would you say that most people in your community are willing to help each other, or that they mostly just look out for themselves?” Participants could answer “1 = try to be helpful”, “2 = try to be somewhat helpful”, “3 = neither”, “4 = somewhat look out for themselves”, or “5 = just look out for themselves”. Responses were dichotomized with the three first alternatives and the two latter alternatives for the analysis: mistrust (responses 4 and 5) and lack of reciprocity (responses 4 and 5). Community-level social capital was obtained by aggregating individual responses and calculating the proportion of subjects reporting mistrust or lack of reciprocity. There has been recent debate as to whether the domain of trust belongs to the concept of social capital [28–30]. We argue, however, that according to the definition of social capital (“the resources generated via social network connections”), trust can be viewed as a crucial “moral resource” which lubricates social exchanges between actors—e.g. reciprocity exchanges as well as the enforcement of norms. In our study, the correlation between social trust and reciprocity was 0.34 at the individual level, and 0.68 at the community level.

#### Psychological distress

Psychological distress was assessed using the Japanese version of the Kessler Psychological Distress scale (K6), comprising six questions on depression and anxiety [31]. Each question was measured on a 5-point scale and the total score ranged from 6 to 30. We set the cut-off at ≥11 to generate a dichotomous variable in line with previous studies on the Japanese population [32].

#### Covariates

We considered the following variables as relevant confounders in the analyses: age (continuous), sex, educational attainment (junior high school, high school, some college or more), frequency of alcohol consumption (none, 1–3 times/month, 1–6 times/week, everyday), smoking status (never/former vs. current), body mass index (less than 25 kg/m^2^, 25 kg/m^2^ or larger), marital status (cohabiting, widowed, divorced, separated, unmarried), socioeconomic status (high, some high, middle, some low, low), and number of cohabiters (one person, two persons, three persons, four persons or more). Socioeconomic status was assessed by a visual analogue scale subjectively and divided into five categories as according to the distributions (1–2 = high, 3–4 = some high, 5 = middle, 6–7 = some low, 8–9 = low).

Social support was measured by the Measurement of Social Support-Elderly (MOSS-E) [33]. The MOSS-E consists of 10 yes or no (Yes/No = 1/0) items comprised of three sub-scales: instrumental support, emotional support, and providing support to others. Of the three sub-scales, we included instrumental support (items 1–3: shopping, cleaning and cooking, and offering to run errands) and emotional support (items 4–6: reassurance, support, and assisting) (see S1 Table for list of items). Regarding emotional support, we did not adopt an item originally included as the seventh item (“Is there someone who is concerned about you and is interested in your welfare?”) as it was replaced by another item concerning visits from commissioned welfare volunteers. Responses were summed to obtain scores for instrumental support (SS1–3) and emotional support (SS4–6). The two sub-categories had good internal consistency reliability (Cronbach’s alphas = 0.85 for both sub-scales).

### Statistical analysis

After excluding subjects with missing values, we conducted a two-level Poisson regression analysis on 9,761 individuals (level 1) nested within 35 communities (level 2). We calculated risk ratios (RRs) and 95% confidence intervals (CIs) for psychological distress. First, we examined the community-level variance in psychological distress without including any explanatory variables (null model). Second, we examined the relationship between individual-level social capital and psychological distress by adjusting for other individual-level covariates (Model 1). Third, we included community-level social capital instead of individual-level social capital (Model 2). Fourth, we adjusted both individual- and community-level social capital variables simultaneously (Model 3). Fifth, social support was included (Model 4). Finally, we adjusted for emotional support and instrumental support instead of social support (Model 5). In Models 3 to 5, individual-level mistrust and lack of reciprocity were group-mean centered to avoid the problem of collinearity between individual- and community-level social capital [6]. With regard to community-level social capital, we estimated RRs per 10% increase of the proportion of mistrust or lack of reciprocity in that community. Furthermore, we tested the statistical cross-level interaction between individual- and community-level social capital.

We considered p-values of less than 0.05 (two-sided test) to be statistically significant. We reported community-level variance and Akaike Information Criteria (AIC) to compare the goodness-of-fit of models. All analyses were performed using STATA 12.1 (StataCorp, College Station, TX, USA).

## Results

Among the 9,761 study subjects, 3,889 (39.8%) reported psychological distress, as shown in Table 1. Psychological distress was more prevalent among subjects reporting mistrust and lack of reciprocity (53.8% and 47.3%, respectively). Table 2 shows the mean proportions for reporting mistrust and lack of reciprocity within the 35 communities (7.4 ± 2.06% and 24.1 ± 4.00%, respectively).

**Table 1:** Characteristics of the study subjects, Okayama, Japan (2010).

			Psychological distress^a^	
Characteristics	No.	%	No.	%
All	9761	100	3889	39.8
Social capital				
Mistrust				
Yes	699	7.2	376	53.8
No	9062	92.8	3513	38.8
Lack of reciprocity				
Yes	2262	23.2	1069	47.3
No	7499	76.8	2820	37.6
Age (Means, SD)	76.6	7.22	77.9	7.42
Sex				
Women	5819	59.6	2469	42.4
Men	3942	40.4	1420	36.0
Educational attainment				
Junior high school	4263	43.7	1969	46.2
High school	4125	42.3	1443	35.0
Some college or more	901	9.2	272	30.2
Missing	472	4.8	205	43.4
Frequency of alcohol consumption				
None	5473	56.1	2395	43.8
1–3 times/month	1128	11.6	398	35.3
1–6 times/week	1373	14.1	491	35.8
Everyday	1493	15.3	473	31.7
Missing	294	3.0	132	44.9
Smoking status				
Never/former	8408	86.1	3351	39.9
Current	777	8.0	280	36.0
Missing	576	5.9	258	44.8
Body mass index (kg/m^2^)				
less than 25	7738	79.3	3106	40.1
25 or larger	1633	16.7	606	37.1
Missing	390	4.0	177	45.4
Marital status				
Cohabiting	6121	62.7	2234	36.5
Bereaved	2863	29.3	1292	45.1
Divorced	159	1.6	62	39.0
Separated	189	1.9	105	55.6
Unmarried	94	1.0	41	43.6
Missing	335	3.4	155	46.3
Socioeconomic status				
Low	1527	15.6	836	54.8
Some low	2104	21.6	940	44.7
Middle	4681	48.0	1645	35.1
Some high	777	8.0	242	31.2
High	205	2.1	54	26.3
Missing	467	4.8	172	36.8
Number of cohabiters (Means, SD)	2.94	2.00	2.86	1.98
Social support^b^ (Means, SD)	4.93	1.87	4.72	1.96
Emotional support	2.59	0.86	2.50	0.92
Instrumental support	2.59	0.86	2.44	0.97

SD: standard deviation.

^a^Psychological distress denotes K6 scores ≥11.

^b^Social support denotes the number of “people who help you” (items 1 to 6).

**Table 2:** Community-level social capital in 35 communities, Okayama, Japan (2010).

	Mean	SD	Range
Mistrust^a^ (%)	7.4	2.06	3.9–12.4
Lack of reciprocity^b^ (%)	24.1	4.00	15.3–33.7

SD: standard deviation.

^a^Community-level mistrust defined as the proportion of residents reporting mistrust within the community.

^b^Community-level lack of reciprocity was defined as the proportion of residents reporting lack of reciprocity within the community.

In the null model, the community-level variance was 0.002 (SE: 0.003) which suggests there was little variation between communities in psychological distress (Tables 3 and 4). When we examined the relationship between individual-level mistrust or lack of reciprocity and psychological distress in Model 1, we found that both individual-level mistrust and lack of reciprocity were associated with a higher likelihood of reporting psychological distress. Likewise, we found positive associations between community-level mistrust or lack of reciprocity and psychological distress in Model 2. When we adjusted for both community- and individual-level social capital simultaneously, the effects of community-level mistrust and lack of reciprocity did not change substantially (Model 3). AIC values of Model 5 in Tables 3 and 4 were smaller than other models, suggesting good model fit. In addition, the cross-level interaction was not statistically significant (mistrust: p = 0.962).

**Table 3:** Risk ratios for psychological distress^a^ associated with mistrust among the elderly, Okayama, Japan (2010).

	Null model		Model 1^b^		Model 2^b^		Model 3^b^		Model 4^b^		Model 5^b^	
			RR	(95% CI)	RR	(95% CI)	RR	(95% CI)	RR	(95% CI)	RR	(95% CI)
*Individual-level*												
Mistrust^c^ (vs. trust)			1.33	(1.18–1.50)			1.32	(1.17–1.49)	1.29	(1.14–1.46)	1.24	(1.09–1.41)
Social support^d^									1.04	(1.02–1.06)		
Emotional support^d^											1.17	(1.11–1.23)
Instrumental support^d^											0.97	(0.92–1.03)
*Community-level*												
Mistrust^e^					1.26	(1.03–1.55)	1.26	(1.03–1.55)	1.23	(1.01–1.51)	1.27	(1.03–1.57)
Community-level variance (SE)	0.002	(0.003)	0	(0.000)	0	(0.000)	0	(0.000)	0	(0.000)	0	(0.000)
AIC	14939		11613		11628		11611		11598		11057	

AIC: Akaike Information Criteria; CI: confidence interval; RR: risk ratio; SE: standard error.

^a^Psychological distress denotes K6 scores ≥11.

^b^Adjusted for age, sex, educational attainment, frequency of alcohol consumption, smoking status, body mass index, marital status, socioeconomic status, and number of cohabiters.

^c^Individual-level mistrust was group-mean centered in Models 3–5.

^d^Per 1-person decrease in the number of “people who help you”.

^e^Community-level mistrust was defined as the proportion of residents reporting mistrust within the community. We show RRs per 10% increase of the proportion of mistrust in the community.

**Table 4:** Risk ratios for psychological distress^a^ associated with lack of reciprocity among the elderly, Okayama, Japan (2010).

	Null model		Model 1^b^		Model 2^b^		Model 3^b^		Model 4^b^		Model 5^b^	
			RR	(95% CI)	RR	(95% CI)	RR	(95% CI)	RR	(95% CI)	RR	(95% CI)
*Individual-level*												
Lack of reciprocity^c^ (vs. reciprocity)			1.24	(1.15–1.34)			1.23	(1.14–1.33)	1.21	(1.12–1.31)	1.18	(1.08–1.28)
Social support^d^									1.04	(1.02–1.06)		
Emotional support^d^											1.16	(1.10–1.23)
Instrumental support^d^											0.97	(0.92–1.03)
*Community-level*												
Lack of reciprocity^e^					1.13	(1.03–1.25)	1.13	(1.03–1.25)	1.12	(1.02–1.24)	1.14	(1.03–1.26)
Community-level variance (SE)	0.002	(0.003)	0	(0.000)	0	(0.000)	0	(0.000)	0	(0.000)	0	(0.000)
AIC	14939		11606		11627		11603		11590		11051	

AIC: Akaike Information Criteria; CI: confidence interval; RR: risk ratio; SE: standard error.

^a^Psychological distress denotes K6 scores ≥11.

^b^Adjusted for age, sex, educational attainment, frequency of alcohol consumption, smoking status, body mass index, marital status, socioeconomic status, and number of cohabiters.

^c^Individual-level lack of reciprocity was group-mean centered in Models 3–5.

^d^Per 1-person decrease in the number of “people who help you”.

^e^Community-level lack of reciprocity was defined as the proportion of residents reporting lack of reciprocity within the community. We show RRs per 10% increase of the proportion of lack of reciprocity in the community.

When we adjusted for social support (Model 4), the coefficients for community-level mistrust and individual-level social capital were somewhat attenuated. The adjusted RRs per 10% increase of the proportion of mistrust and lack of reciprocity in the communities were 1.23 (95% CI: 1.01–1.51) and 1.12 (95% CI: 1.02–1.24), respectively. Social support for each 1-person decrease in the number of “people who help you” was associated with a higher likelihood of psychological distress. When we adjusted for emotional support and instrumental support (Model 5), we found robust associations for community-level social capital and positive associations for emotional support only. No clear associations were found for instrumental support. In addition, the cross-level interaction was not statistically significant (lack of reciprocity: p = 0.715).

## Discussion

The present study suggests that community-level mistrust and lack of reciprocity are associated with psychological distress among Japanese elderly people. These results did not change substantially even after simultaneously adjusting for individual-level perceptions of social capital and social support.

It is notable that associations between community-level social capital and psychological distress were independent of social support and individual-level social capital. Even after adjusting for social support and/or individual social capital, we found robust associations with regard to community-level social capital. Furthermore, we also observed that the RRs of individual perceptions of social capital did not change substantially after controlling for social support. These results imply that the concept of community/individual social capital is not serving as a mere proxy for social support and that community social capital has validity as a community contextual influence on mental health. In addition, the point estimates of community- and individual-level social capital were greater than that of social support, implying the significance of examining social capital at both individual and ecological levels. Although it has been argued that the pathways from social capital to psychological distress are via supportive relationships [6], further studies are required to clarify the relationship between social capital and social support relative to psychological distress.

Previous evidence supports the view that higher community-level trust is associated with a lower likelihood of reporting psychological distress [10, 12, 34, 35]. In the present study, as the proportion of those reporting mistrust or lack of reciprocity rises by 10% in the community, the likelihood of psychological distress increases by 1.23 and 1.12 times, respectively. The locale of the present study consisted of rural communities, and older residents of Okayama tend to be more residentially stable compared to other major metropolitan areas in Japan (e.g. Tokyo). In this context, older residents seem to have built their informal social control over deviant health-related behavior, making it easier to obey norms and reduced unnecessary conflicts for them. Thus, community-level social capital could have a strong impact on health among the elderly because their spatial scale of collective socialization tends to be more restricted and local compared to working age adults or school-aged children.

Our findings with respect to community-level lack of reciprocity show lower point estimates than community-level mistrust. This difference is probably attributable to measuring different aspects of social capital. Reciprocity is the willingness to help neighbors with the expectation that the favor will be returned in the future [36]. Since the *Edo* period (1600 to 1868) in Japan, reciprocity has been encapsulated by the concept of *Muko-sangen ryo-donari*–which translates as “the three houses opposite and one on either side of one’s home”, representing the social distances which forms the basis of neighborly gift exchange, greetings, and mutual aid. It implies greater mutual connections with neighbors rather than trust. However, such connections with neighbors are less common now than in the past, even among the elderly, because of the rise in the number of nuclear families, and women’s labor force participation [37]. On the other hand, trust in the community may reflect relationships developed over an extended period of time. Indeed, we found that the proportion of residents reporting lack of reciprocity (24.1%) was higher than that of mistrust (7.4%) (Table 2). Thus, reciprocity may impact less on an older person’s health than trust in Japan.

Several limitations should also be noted within our study. First, there is the possibility that we did not capture the appropriate boundaries based on the natural social ties among residents. Thus, the use of administrative boundaries may result in the misclassification of the areal unit and inaccurate estimations of the community-level social capital [38]. Further studies may benefit from spatial analysis that recognizes a spatial continuum such as spillover effects from adjacent neighbors. Second, both exposure and outcome were assessed by self-reported questionnaires. If individuals who report psychological distress also to report a low level of social capital, then this raises the possibility of common method bias. However, even if individual questions were based on perception, once these were aggregated to the community level, then these indicators did not only measure subjective aspects. Therefore, it is unlikely that our results were substantially influenced by common method bias. Third, care must be taken when generalizing our results because of the limited study samples in rural areas, despite relatively good (65.6%) response rate. Lastly, the possibility of reverse causation cannot be ignored because of the cross-sectional design of our study.

## Conclusions

The present study shows that a lack of community-level social capital is associated with psychological distress among the Japanese elderly population, even after simultaneously adjusting for individual-level social capital and social support. Although we cannot exclude reverse causation because of the cross-sectional design, our findings do contribute to further understand the relationship between social capital and mental health and the promotion of future policies on mental health to address the aging population. Future research should explore the robustness of our findings via a longitudinal design.

## Supporting Information

S1 Table The Measurement of Social Support-Elderly (MOSS-E). (PDF)
