# Correction: Community-Level Social Capital and Psychological Distress among the Elderly in Japan: A Population-Based Study

**DOI:** 10.1371/journal.pone.0144683

**Published:** 2015-12-11

**Authors:** Tomoko Kobayashi, Etsuji Suzuki, Masayuki Noguchi, Ichiro Kawachi, Soshi Takao

The use of the term “risk ratio” is incorrect throughout the manuscript. The correct term should be “rate ratio.”
