# Supplementary material for: Community-Level Social Capital and Psychological Distress among the Elderly in Japan: A Population-Based Study
Source: PLoS One. 2015 Nov 9;10(11):e0142629. doi: 10.1371/journal.pone.0142629 (PMC4638358; doi:10.1371/journal.pone.0142629)
Supplement: S1 Table — (PDF) [file pone.0142629.s001.pdf]

Supplementary Table

The Measurement of Social Support-Elderly (MOSS-E).

1.	Is there anyone you can ask to help you with cooking and shopping four your daily needs?
2.	Is there anyone you can ask to help you with your gardening, cleaning, washing etc.?
3.	Is there anyone you can easily ask to help you with other chores?
4.	Is there a person who cares for you and by your side when you are in difficulty?
5.	Is there someone you can talk to or ask for advice when you are worried?
6.	Is there someone who encourages you when you are down or feeling depressed?
